# Phosphotyrosine Substrate Sequence Motifs for Dual Specificity Phosphatases

**DOI:** 10.1371/journal.pone.0134984

**Published:** 2015-08-24

**Authors:** Bryan M. Zhao, Sarah L. Keasey, Joseph E. Tropea, George T. Lountos, Beverly K. Dyas, Scott Cherry, Sreejith Raran-Kurussi, David S. Waugh, Robert G. Ulrich

**Affiliations:** 1 Molecular and Translational Sciences Division, U.S. Army Medical Research Institute of Infectious Diseases, Frederick, MD, 21702, United States of America; 2 The Geneva Foundation, Tacoma, WA, 98402, United States of America; 3 Macromolecular Crystallography Laboratory, Center for Cancer Research, National Cancer Institute, Frederick, Maryland, 21702, United States of America; 4 Basic Science Program, Leidos Biomedical Research, Inc., Frederick National Laboratory for Cancer Research, Frederick, Maryland, 21702, United States of America; Pacific Northwest National Laboratory, UNITED STATES

## Abstract

Protein tyrosine phosphatases dephosphorylate tyrosine residues of proteins, whereas, dual specificity phosphatases (DUSPs) are a subgroup of protein tyrosine phosphatases that dephosphorylate not only Tyr(P) residue, but also the Ser(P) and Thr(P) residues of proteins. The DUSPs are linked to the regulation of many cellular functions and signaling pathways. Though many cellular targets of DUSPs are known, the relationship between catalytic activity and substrate specificity is poorly defined. We investigated the interactions of peptide substrates with select DUSPs of four types: MAP kinases (DUSP1 and DUSP7), atypical (DUSP3, DUSP14, DUSP22 and DUSP27), viral (variola VH1), and Cdc25 (A-C). Phosphatase recognition sites were experimentally determined by measuring dephosphorylation of 6,218 microarrayed Tyr(P) peptides representing confirmed and theoretical phosphorylation motifs from the cellular proteome. A broad continuum of dephosphorylation was observed across the microarrayed peptide substrates for all phosphatases, suggesting a complex relationship between substrate sequence recognition and optimal activity. Further analysis of peptide dephosphorylation by hierarchical clustering indicated that DUSPs could be organized by substrate sequence motifs, and peptide-specificities by phylogenetic relationships among the catalytic domains. The most highly dephosphorylated peptides represented proteins from 29 cell-signaling pathways, greatly expanding the list of potential targets of DUSPs. These newly identified DUSP substrates will be important for examining structure-activity relationships with physiologically relevant targets.

## Introduction

Tyrosine Tyr(P) phosphorylation (Tyr(P)) is a frequent and reversible protein modification that triggers essential molecular interactions, enzyme activation, changes in signaling pathways and many other key cellular events. Proteins modified during these complex biochemical cycles are in turn dephosphorylated by diverse protein tyrosine phosphatases (PTPs). Dual-specificity phosphatases (DUSPs), first exemplified by the VH1 phosphatase of pox viruses [1], are a specialized subset of PTPs that hydrolyze phosphate groups of tyrosine and serine or threonine residues of proteins involved in the regulation cell growth, proliferation, apoptosis, migration and innate immunity [2, 3]. The human genome encodes approximately 61 DUSPs that cluster into seven homology groups [2], while variations in regulatory domains and binding partners guide the functional diversity of each enzyme. The regulatory domains may include nuclear localization sequences, kinase interaction modules, nuclear export signals, and Cdc25/rhodanese-homology domain motifs. Catalytic domains are conserved in all DUSPs and harbor the unique consensus sequence motif (H/V)CX_5_R(S/T), where X denotes any amino acid residue [4]. The conserved active site residues cysteine and arginine contained within the ‘P-loop’ motif are critical for substrate binding and catalysis [5]. Because of their pivotal role in phosphorylation-dependent cellular pathways, modulation of DUSP activities may have a therapeutic effect in many chronic or infectious human diseases [2, 3, 6–8]. Considering the potential medical benefits of selectively targeting DUSPs, progress in developing therapeutic drugs has been slow. The characterization of biologically-relevant substrates that can be incorporated into screening assays will serve to hasten progress in drug development.

The mitogen-activated protein (MAP) kinase phosphatases (MKPs) are DUSPs that dephosphorylate the MAP kinase signaling pathways proteins of ERK1/2, p38s, and JNKs [9, 10]. The 11 MKPs, which include DUSP1 and DUSP7, contain a MAPK binding domain (MKB) in addition to the protein tyrosine phosphatase (PTP) catalytic domain [6], whereas there are 19 atypical and low molecular weight DUSPs that lack the MKB domain [6]. Examples of atypical DUSPs are DUSP3, 14, 22 and 27. The MKPs and atypical DUSPs dephosphorylate both Thr(P) and Tyr(P) residues within the MAPK activation motif Thr-Xaa-Tyr and exert distinct signals and functions through temporal, spatial and substrate selectivity [11]. For example, both DUSP3 (also known as VHR) and DUSP1, the first mammalian DUSP identified [12], dephosphorylate ERK1/2, p38s, and JNKs but differ in subcellular localization [11]. DUSP3 dephosphorylates ERK1/2, p38 and JNKs [13, 14], while DUSP22 serves as a positive regulator of the MAPK-signaling pathway by dephosphorylation of JNK [15]. In addition to the cellular substrate specificity, many DUSPs also regulate specific signaling pathways and cellular processes. For example, DUSP14 negatively regulates NFκ-B activation by dephosphorylating TAK1 at Thr-187 [16], and DUSP22 is required for full activation of JNK signaling pathway through a mechanism that increases the activation of the upstream JNK kinases MKK4 and MKK7 [17, 18]. Further, DUSP27, which is expressed in skeletal muscle, liver and adipose tissue, was implicated in energy metabolism [19]. The Cdc25 isoforms A-C, which are important regulators of the cyclin-dependent kinases, hydrolyze Tyr(P) or Thr(P) residues and belong to a distinct class of cysteine-based PTPs [20]. The C-terminal catalytic domains are highly homologous among all Cdc25 isoforms. The amino acid residues R488 and Y497 were implicated in protein substrate recognition by Cdc25s [21] but are distant from the catalytic site, which is extremely shallow.

There is a considerable gap in our understanding of the structural basis for DUSP substrate specificity. While the catalytic domains share a common protein fold, differences in surface features are likely to influence substrate interactions. The Tyr(P)-mimetic substrates *para*-nitrophenylphosphate (*p*NPP) and 6,8-Difluoro-4-Methylumbelliferyl Phosphate (DiFMUP) are widely employed to examine PTP catalysis, but data from studies using these small chemical compounds provide little information about enzyme specificity. Compared to small molecule substrates, phosphorylated peptides present many advantages, such as ease of synthesis and modification, and are more physiologically relevant targets. In this study, we used a microarrayed library comprised of > 6000 Tyr(P) peptides to identify substrate recognition motifs of the isolated catalytic domains from ten DUSPs, and further analyze interactions of DUSP substrate-trapping mutants with intact cellular proteins.

## Materials and Methods

### Materials

Anti-Tyr(P) specific mouse monoclonal antibody P-Tyr-100 was purchased from Cell Signaling Technology (Danvers, MA) and Alexa fluor 647 goat anti-mouse antibody was purchased from Invitrogen Life Technologies., Inc., (Grand Island, NY). The small molecule substrate *p*NPP was purchased from EMD Millipore (Billerica, MA) and remaining chemicals were purchased from Sigma-Aldrich (St. Louis, MO).

### Recombinant Protein Expression and Purification

The following full length or catalytic domains of human DUSP1, DUSP3, DUSP7, DUSP22, Cdc25A, Cdc25A and Cdc25B were all expressed as maltose binding protein (MBP) fusion proteins, cleaved by TEV protease, and purified using the method described by Tropea et al [22]. The full-length of variola major H1 (VH1); human DUSP14 and DUSP27 proteins were purified as described previously [23–25]. Purified proteins were resolved in a polyacrylamide gel and visualized by Coomassie Brilliant blue staining. Images were acquired with a ChemiDoc MP imaging system (Bio-Rad, Hercules, CA, USA). Determination of Kcat/Km for each purified protein was performed as described (Hogan et al. submitted).

### Tyr(P) Peptide Microarray Assay

The annotated phosphosites-Tyr-phosphatase microarray slides (PHOS-MA-PY) were purchased from Jerini Peptide Technology (GmbH, Berlin, Germany). Each microarray consisted of three identical subarrays of 16 blocks comprised of 20 rows and 20 columns, resulting in 6218 Tyr(P) peptides printed in triplicate on each glass slide. Human sequences were represented by 5765 peptides while the remainder originated from a variety of organisms. Most of the peptides on the microarray have a length of 13 amino acids, with the Tyr(P) residue in the middle position. The peptide microarray slides were blocked with 1X Fast blocking buffer (Thermo Scientific Inc., Rockford, IL, USA). Spacers were inserted between the peptide microarray and a blank slide, and phosphatases (0.1–1.0 mg/ml) in citrate buffer (pH 6.4) were added from one corner of the slide until the space between the slides was completely filled. The slides were incubated in a humid chamber (22°C) 10–60 min, depending on the phosphatase used, the blank slide was removed and the microarray was washed (3X, 10 min) with TBS-0.1% (v/v) Tween-20. The wet slides were submerged in an anti-Tyr(P) (1:1000) antibody solution for 1 hour (22°C), washed 3X for 10 min with TBS-0.1% (v/v) Tween-20 before submerging in the Alex 635 Goat anti-mouse (1:2000) solution for 1 hour. The microarrays were then washed with TBS-0.1% (v/v) Tween-20 (3X, 10 min) and distilled water (2X, 5 min). Air-dried microarrays were scanned (635nm) using an AXON GENEPIC 4000B scanner (Molecular Devices, Sunnyvale, CA, USA). Similar instrument settings were used to scan all peptide microarray slides. Digital images of the results were analyzed with GenePix Pro 5.1 software (Molecular Devices). Background pixel counts were subtracted from triplicate spots and the results were averaged.

### Microarray Data Analysis

The dephosphorylation status of each peptide on the peptide microarray was obtained by measuring the florescence intensity. Pixel values for each spot on the microarray were subtracted from background and recorded in an excel file as relative florescence units (RFUs). The florescence intensity for each peptide, presented as relative florescence units (RFUs), was calculated by averaging the florescence intensity of triplicate spots for each peptide. The reference control slide was treated with buffer only. Data for a total of 6218 annotated phosphotyrosine peptides (18654 spots per peptide microarray) were collected for the reference slides and DUSP treated slides. Unreliable data from individual peptide spots were removed from further analysis based on the following criteria:

1. Spots with negative RFU
2. $\frac{Median_{RFU635}}{Mean_{RFU635}}$ or $\frac{Mean_{RFU635}}{Meadian_{RFU635}}$ of a spot was > 1.5

The RFUs collected from each DUSP treated phosphotyrosine peptide microarray were combined and quantile normalized using the “preprocessCore” package (http://www.bioconductor.org/packages/release/bioc/html/preprocessCore.html) in R/BioConductor. The percentage dephosphorylation of each peptide was calculated using the following equation:

$$\%\;dephosphorylation=\frac{RFU_{reference}-RFU_{DUSPtreated}}{RFU_{reference}}X\;100\%$$

A subgroup of phosphotyrosine peptides (n = 916) with high fluorescent signals (the relative fluorescent signal greater than 40,000 in the reference slide) were selected for hierarchical cluster analysis. The hierarchical cluster analysis of the microarray data for substrate dephosphorylation was performed with MultiExperiment Viewer (MeV) v4.7.4 [26], using Pearson correlation and Average Linkage Clustering algorithm.

### Phylogenic Analysis

The lengths of the catalytic domains of the DUSP proteins used in this study ranged from 171 amino acids long in VH1 to 380 amino acids long in Cdc25A. Minimal catalytic domain amino acid sequences of around 140 amino acids (S1 Fig) were derived from structural and sequence alignments. Phylogenetic trees were constructed by three different multiple sequence alignment methods (the Jotun Hein Method, the Clustal V method and the Clustal W method) available in the MegAlign sequence analysis software program (DNAStar Inc., Madison, WI). Multiple sequence alignments (MSAs) were constructed by using the conserved active site motifs (HCXXXXXR) for each phosphatase along with 15 flanking amino acids on both ends. CLUSTALW2 [27] was used to generate three MSAs, each using a different gap opening penalty (5, 10, and 25), with BLOSUM62 as the protein weight matrix and all other options left as default. T-Coffee Combine [28, 29] was then used to generate a single alignment that had the best agreement for all of the MSAs. To eliminate poorly aligned positions and divergent regions in the combined alignment, the alignment was filtered using Gblocks [30, 31] with no gap positions within the final blocks, strict flanking positions, and no small final blocks. Gblocks reported a single conserved block starting seven residues upstream of the active site and ending at the conserved arginine residue. This 15 residue region was used to reconstruct a phylogenetic tree using the maximum likelihood method implemented in the PhyML program (v3.0 aLRT) [32]. The BLOSUM62 substitution model was selected and 4 gamma-distributed rate categories to account for rate heterogeneity across sites. The gamma shape parameter was estimated directly from the data (gamma = 0.757). Tree topology and branch length were optimized for the starting tree with subtree pruning and regrafting (SPR) selected for tree improvement. Reliability for internal branches was assessed using a bootstrap method with 1000 replicates.

### Sequence Motif Extraction

Consensus sequence motifs for substrates recognized by each phosphatase were generated by pLogo (http://plogo.uconn.edu/). A total of 6032 unique 13-residue peptides in the peptide microarray library were selected as the whole data set. For each analysis, ~500 peptides with the highest level of dephosphorylation (≥80%) from the peptide microarray were used as the foreground data set with criteria that the original peptides all have signal intensities of RFU >40,000. The background data set was obtained by subtracting the foreground sequences from the whole data set, and statistically-significant residues were calculated by the algorithm. The Tyrosine at position 7 was selected as the fixed position with frequency of 100% for generation of the substrate motif for each DUSP. The Tyr(P) residue of each 13-residue peptide was assigned as the zero position, residues on the N-terminal side of Tyr(P) were assigned from -1 to -6, and residues on the C-terminal side were assigned from +1 to +6.

### Analysis of Signaling Pathways

Human proteins represented by the most active peptide substrates were used for analysis of biological interactions by the Kyoto Encyclopedia of Genes and Genomes (KEGG) pathways data sets. Of the 583 human proteins represented by peptides that exhibited >80% dephosphorylation by any DUSP, 11 did not have a KEGG identifier, while 262 had no pathway associations, resulting in a final list of 310 proteins that were used for the pathway analysis. The substrate proteins were compared to a background consisting of the remaining 1,610 microarrayed peptides that were associated with human KEGG pathways. KEGG pathway associations were filtered to include only those pathways involved in signaling, resulting in 29 pathways for substrate and 33 for background proteins. Chi-square p-values were calculated for each pathway to identify enrichment for substrate proteins, implementing a Bonferroni correction factor for a significance threshold of p<0.001515.

### Protein Structure Models

Superimposed ribbon representations of DUSP3 (PDB: 1VHR), DUSP14 (PDB: 2WGP), DUSP22 (PDB: 1WRM) and Cdc25B (PDB: 1QB0) catalytic domains were generated using PyMOL Molecular Graphics System (Version 1.5.0.4 Schrödinger, LLC.). The PDBeFold online server (http://www.ebi.ac.uk/msd-srv/ssm/) was used for structural homology comparisons. The surface electrostatic potential of the catalytic sites were calculated and models were generated using ICM Browser Pro (MolSoft LLC, San Diego, CA).

## Results

### Recombinant DUSPs

**Fig 1:**
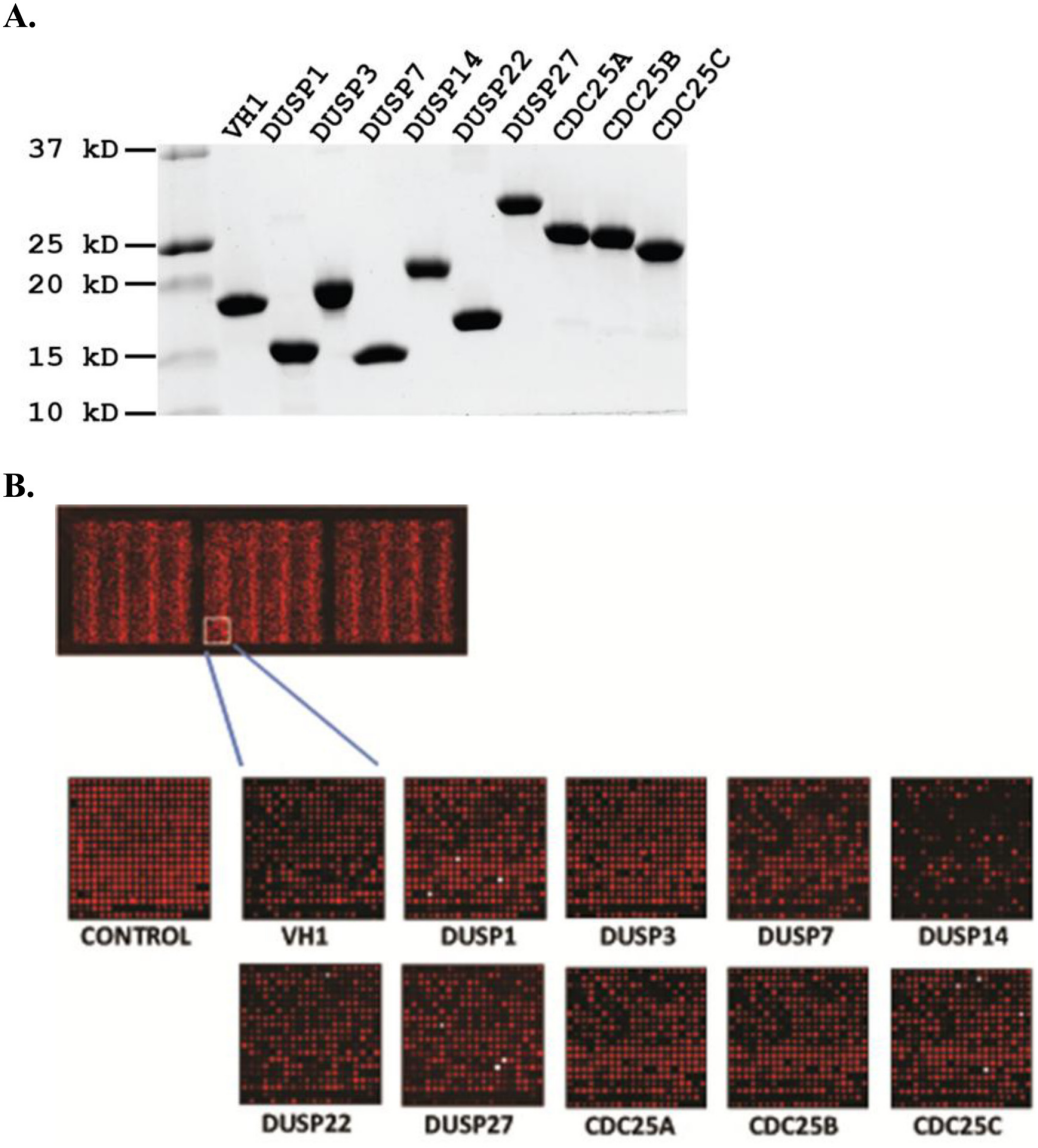
Tyr(P) peptide microarray. (**A**) Coomassie blue staining of a SDS-PAGE gel showing the recombinant DUSP proteins examined. (**B**) Scanned images of DUSP treated Tyr(P) peptide microarrays. The human Tyr(P) peptides (>6000) were microarrayed in three identical subarrays on each slide. The microarrays were incubated with individual DUSPs and remaining Tyr(P) content was measured using anti- Tyr(P) monoclonal antibody and an Alexa-635 secondary (anti-mouse IgG) antibody. The control reference slide was treated with buffer only. The images were obtained from the same area of each slide. Each spot represents one peptide.

The phosphatases used in our study were selected based on diversity of function and for potential involvement in human diseases (Table 1). The full length or catalytic domains of variola virus VH1 [23], human DUSP1 [33], DUSP3 [14], DUSP7 [34], DUSP14 [24], DUSP22 [35], DUSP27 [25], Cdc25A [36], Cdc25B [37] and Cdc25C(unpublished data), were produced in *Escherichia coli* as His-MBP tagged proteins and purified by immobilized metal-affinity chromatography (IMAC) with Ni-NTA resins. His-MBP tags were proteolytically removed using TEV protease and the proteins were further purified using size exclusion chromatography. All recombinant DUSP proteins used in our studies were highly purified (Fig 1A) and stable in solution. DUSP3 had the highest kcat/Km value by *p*-nitrophenyl phosphate (pNPP) assay, and thus the highest enzyme efficiency, while Cdc25A, B and C had the lowest kcat/Km values (Table 1).

**Table 1:** Dual specificity phosphatases examined.

Name	Amino acid residues	Active-site motif												Kcat/Km (M^-1^ s^-1^)	Function and disease association
VH1(2P4D^*a*^)	1–171	P	V	L	V	H	C	V	A	G	V	N	R	198^*b*^	Smallpox [1, 38]
DUSP1		R	V	F	V	H	C	Q	A	G	I	S	R	37^*b*^	Breast cancer, lung cancer, prostate and ovarian cancer [39–43]
DUSP3(1VHR^*a*^)	8–181	R	V	L	V	H	C	R	E	G	Y	S	R	541^*b*^	Cervix carcinoma [44]
DUSP7		G	V	L	V	H	C	L	A	G	I	S	R	10	Acute and myeloid leukemia [45]
DUSP14(2WGP^*a*^)	2–191	A	T	L	V	H	C	A	A	G	V	S	R	181^*b*^	Proliferation of pancreatic *β*-cells [46]
DUSP22(1WRM^*a*^)	1–152	S	C	L	V	H	C	L	A	G	V	S	R	346^*b*^	Breast cancer, lymphomas, Alzheimer’s disease [47–50]
DUSP27(2Y96^*a*^)	2–220	K	I	L	V	H	C	V	M	G	R	S	R	93	unknown
Cdc25A(1C25^*a*^)	335–491	I	I	V	F	H	C	E	F	S	S	E	R	1^*b*^	Cell cycle, cancer [51, 52]
Cdc25B(1QB0^*a*^)	374–551	I	V	V	F	H	C	E	F	S	S	E	R	28^*b*^	Cell cycle, cancer, viral infection [51–53]
Cdc25(C3OP3^*a*^)	280–446	I	L	I	F	H	C	E	F	S	S	E	R	14^*b*^	Cell cycle, cancer [51]

^*a*^ Protein Data Base code

^*b*^ Hogan, M. et al., 2014 (submitted)

### Dephosphorylation of Microarrayed Tyr(P) Peptides

**Fig 2:**
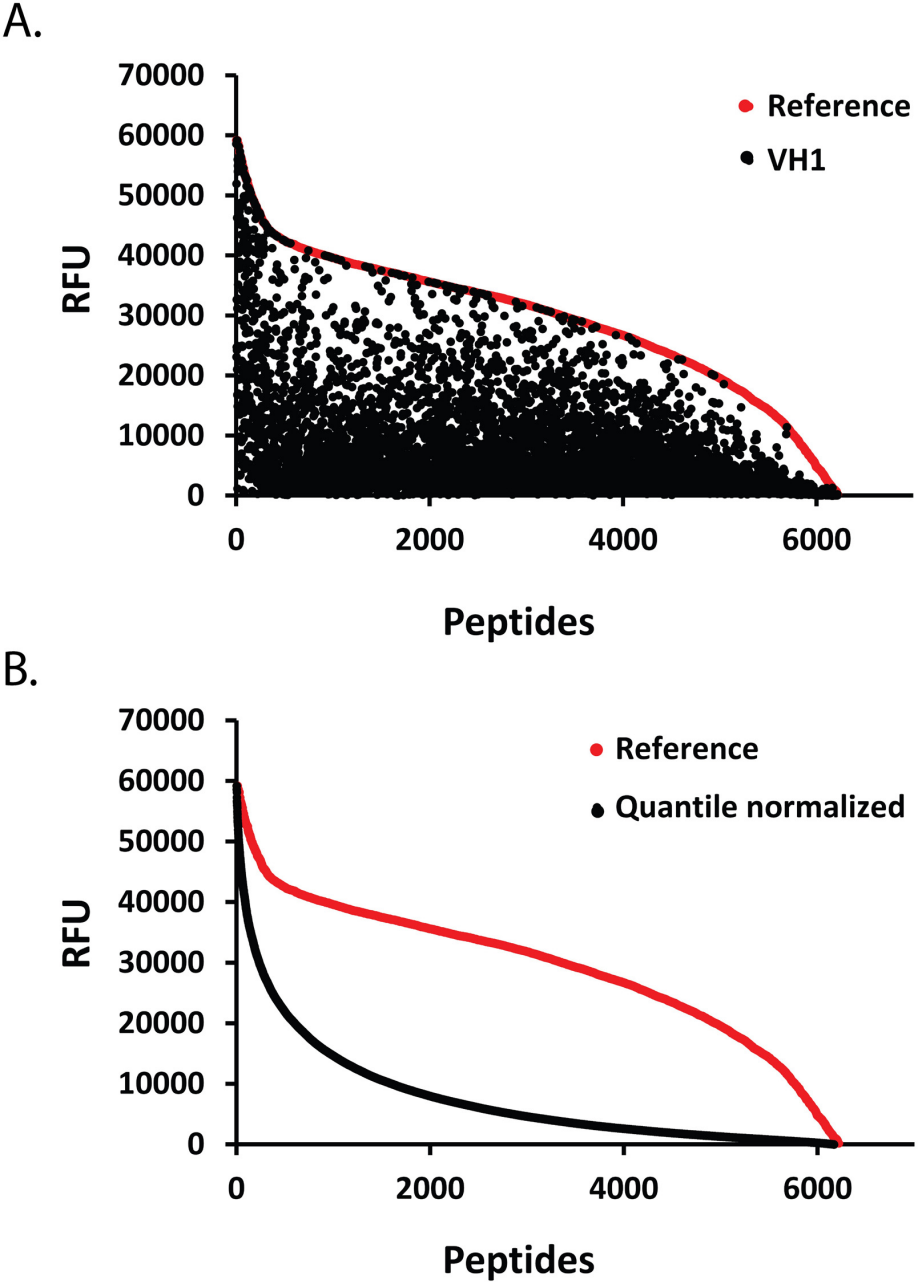
Distribution of dephosphorylation data. (**A**) Representative results of Tyr(P) dephosphorylation of the peptide microarray library by VH1, the poxvirus DUSP. The scatter plot shows the relative florescence units (RFU) of the reference peptide microarray (red dots) ranked from high to low, and the RFUs for corresponding VH1-treated peptides (black dots). Each dot represents the average of triplicate values for one peptide in the library. (**B**) Untreated reference peptide microarray (red dots) compared with an overlay of quantile normalized results for each DUSP showing Tyr(P) peptide dephosphorylation (black dots). Peptides in each data set were sorted from high to low based on RFU.

We used microarrays of Tyr(P) peptides as a high-throughput method to identify substrates for each DUSP. The microarrays consisted of >6000 Tyr(P) peptides comprising known phosphorylation sites (JPT, Berlin, Germany). The 13-residue peptides were synthesized with the Tyr(P) residue in the center, flanked by six residues of each unique protein sequence, and covalently immobilized in triplicate on glass slides via the N-terminus (Fig 1B). Peptide dephosphorylation was assessed by incubating the microarray surface with anti-Tyr(P) antibody, followed by a goat anti-mouse IgG, conjugated to Alexa-647. The experimental conditions were empirically optimized by varying the incubation time and the amount of phosphatase added to the peptide microarray slides to obtain Tyr(P) peptides dephosphorylation data that could be compared among all DUSPs. Digital images of fluorescent-signal intensities representing dephosphorylation were collected by a laser scanner and used for data analysis. Because peptide recognition by the anti-Tyr(P) antibody was potentially affected by sequence context [54], dephosphorylation data were referenced to peptide microarrays treated with buffer only (no DUSP) to compensate for any sequence-specific variability. Fig 1B presents an image of Tyr(P) dephosphorylation by each DUSP for a subset of peptides. As shown by the example of VH1 in Fig 2A, the extent of DUSP dephosphorylation varied considerably by peptide, and this pattern was unique for each DUSP. The microarray data presented a broad continuum of dephosphorylation across the microarrayed substrates for all phosphatases (Fig 2B), suggesting both positive and negative contributions of each peptide residue. Further, the distribution of microarray dephosphorylation data from high to low peptide signal intensity was the same for each phosphatase (Fig 2B), indicating equivalency for experimental conditions.

### Amino Acid Sequence Motifs for DUSP Substrates

**Fig 3:**
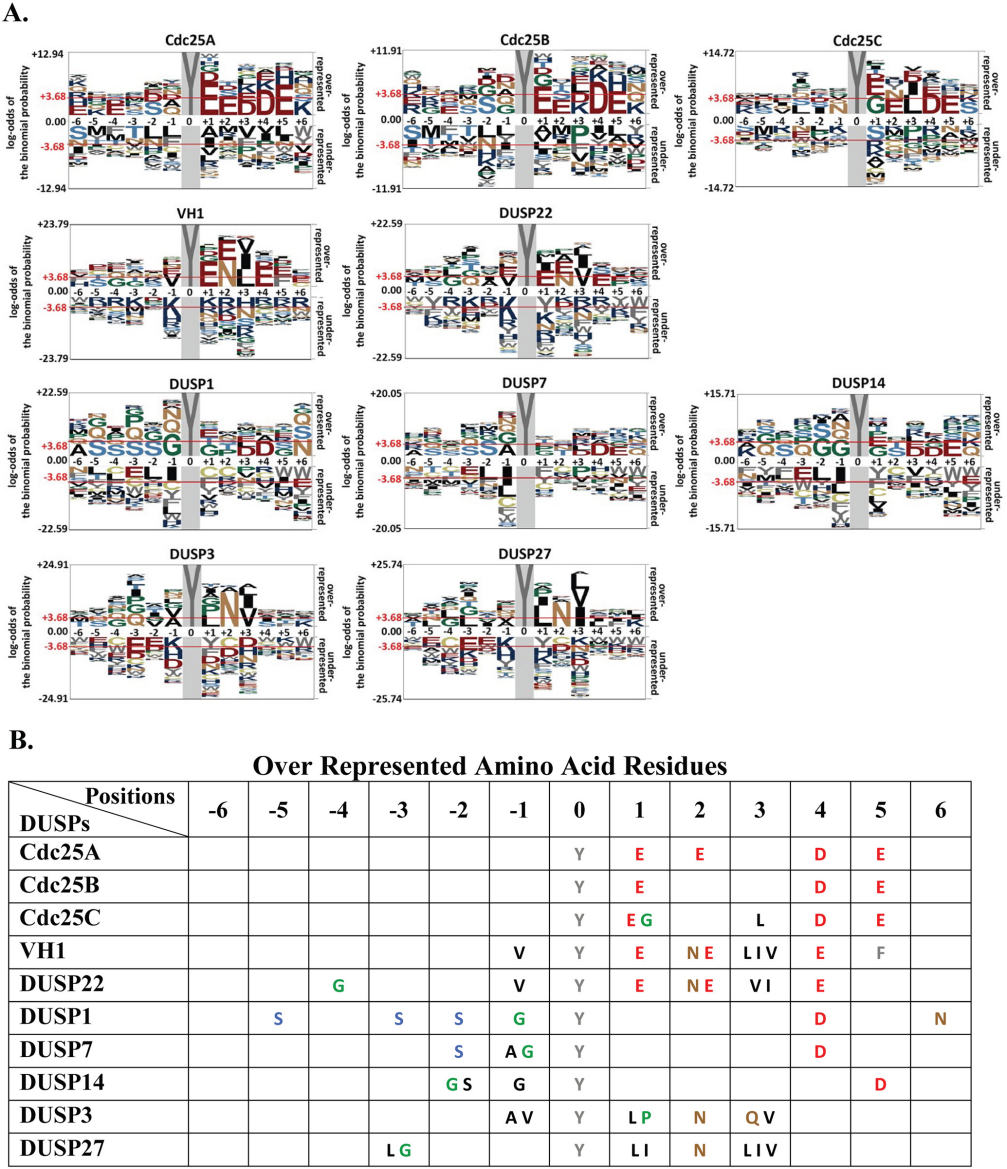
Substrate sequence motifs for each DUSP. (**A**) Results (pLogo) were derived using the 500 most dephosphorylated peptides as foreground (n = 500) and all other peptides in peptide library as background set (n = 5532) peptides sequences for each DUSP protein. Over-represented amino acid residues are above and under-represented amino acid residues are below the x-axis. The height of each single letter represents the statistical significance of the amino acid at that position. The horizontal red lines above and below the x axis correspond to Bonferroni-corrected statistical significance values (p ≤ 0.05). Hydrophobic amino acids (A, I, L, V and M), black; acidic amino acids (D and E), red; basic amino acids (R, H and K), blue; neutral amino acids (Q and N), brown; aromatic amino acids (F, W and Y), gray; and polar amino acids (T and S), light blue. Special amino acids G and P are colored in green and C are colored in dark Khaki. Zero position in the center of the peptide sequence represents the Tyr(P) residue in all motif logos. (**B**) Statistically significant residues that were over-represented at each position are listed for each DUSP.

To determine the sequence motif recognized by each DUSP, we used pLogo [55] to compare the residue frequency in the most dephosphorylated peptide data set and the residue frequency in the background data set in a position-specific manner. The conserved substrate motifs for each DUSP were generated by a graphical representation (pLogo) of the patterns within a multiple sequence alignment residue in which the residue heights are scaled relative to their statistical significance [55]. Although each motif was unique, two general trends in substrate recognition were evident (Fig 3A and 3B). For the first class of substrate motifs, the negatively charged amino acid residues Asp (D) and Glu (E) dominated the overrepresented residues for Cdc25s, VH1 and DUSP22 (Fig 3A and 3B) in at least 3 positions, while neutral Gly (G) and polar Ser (S), were overrepresented residues for DUSP1, DUSP7, DUSP14, DUSP3 and DUSP27 (Fig 3A and 3B). For the second class of substrate motifs (Fig 3A and 3B), DUSP3 and DUSP27, DUSP1, DUSP7 and DUSP14 preferred non-charged residues around the Tyr(P) residue. For DUSP3 and DUSP27, negatively-charged residues were underrepresented at all positions (Fig 3A and 3B), while overall the positively charged amino acid residues Lys (K), Arg (R), and His (H) were rarely observed in any of the motifs. Further, VH1, DUSP22, DUSP3 and DUSP27 preferred Asn at position 2 and Val at position 3. We note that a report by Kohn and coworkers concluded that VHR has a preference for glutamic acid at the -1 position of the target dephosphorylation site, whereas our results showed that alanine and valine have a high frequency occurrence at the -1 position of VHR [56]. The discrepancy could be due to differences in experimental methods and substrates employed. In contrast to the known MAPK activation motif (Thr-Xaa-Tyr), a Ser residue dominated the -2 position for DUSP1, DUSP7 and DUSP14 substrate motifs (Fig 3B), perhaps suggesting that only the phosphorylated Thr residue is favored in the -2 position.

### Relationship between Peptide Substrate and Cell-Signaling Pathways

**Fig 4:**
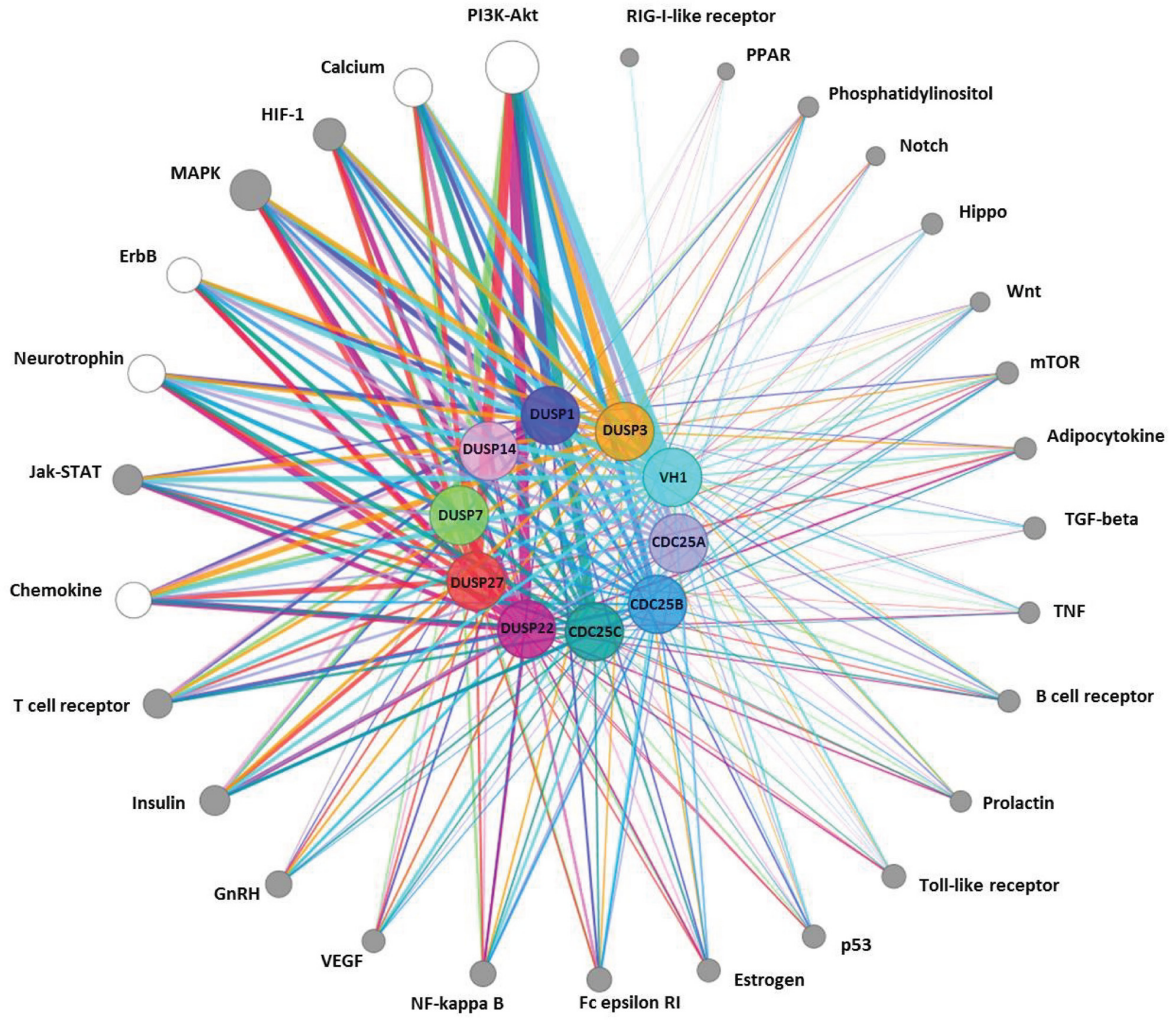
Cell signaling pathways represented by highly dephosphorylated peptides. A total of 29 signaling pathways were identified in the subset of significant peptides recognized by a group of 10 phosphatases. Each phosphatase, as well as its corresponding edges, is color coded. Pathway node size corresponds to number of peptides belonging to that pathway cluster, while edge weight corresponds to the number of peptides in the pathway cluster recognized by the phosphatase. Pathway nodes that are over-represented when compared to a background list encompassing all peptides on the microarray (p<0.001515) are shown in white, while all other pathway nodes are grey.

We selected the most active peptide substrates (696 with >80% dephosphorylation by any DUSP) to examine associations between specific substrates and cell-signaling pathways. Approximately 53% (310) of the 583 human proteins represented by the selected Tyr(P) peptides, were mapped to 29 KEGG signaling pathways [57, 58], as shown in Fig 4. Complexity of the pathway clusters varied from one protein involved in the PPAR signaling pathway to 34 proteins mapped to the PI3K-Akt pathway, and some peptides were found in more than one cluster. Each phosphatase connected to at least 25 clusters, while some pathways had few connections to the enzymes. For example, the two proteins of the RIG-I-like receptor signaling pathway cluster were only targeted by VH1, while the Notch signaling pathway cluster, containing 3 proteins, was targeted by VH1, DUSP3, DUSP22, DUSP27, and Cdc25C. The signaling pathways of PI3K-Akt, calcium, ErbB, neurotrophin, and chemokines (Fig 4; S1 Table) were significantly enriched (p <0.001515, with Bonferroni correction) by comparison to a background list of proteins representative of all peptides printed on the microarray, while MAPK, HIF-1, Insulin, Jak-STAT, and NF-κB pathways were marginally enriched (p ≤0.05).

### Relationship between Peptide Substrates and DUSPs

**Fig 5:**
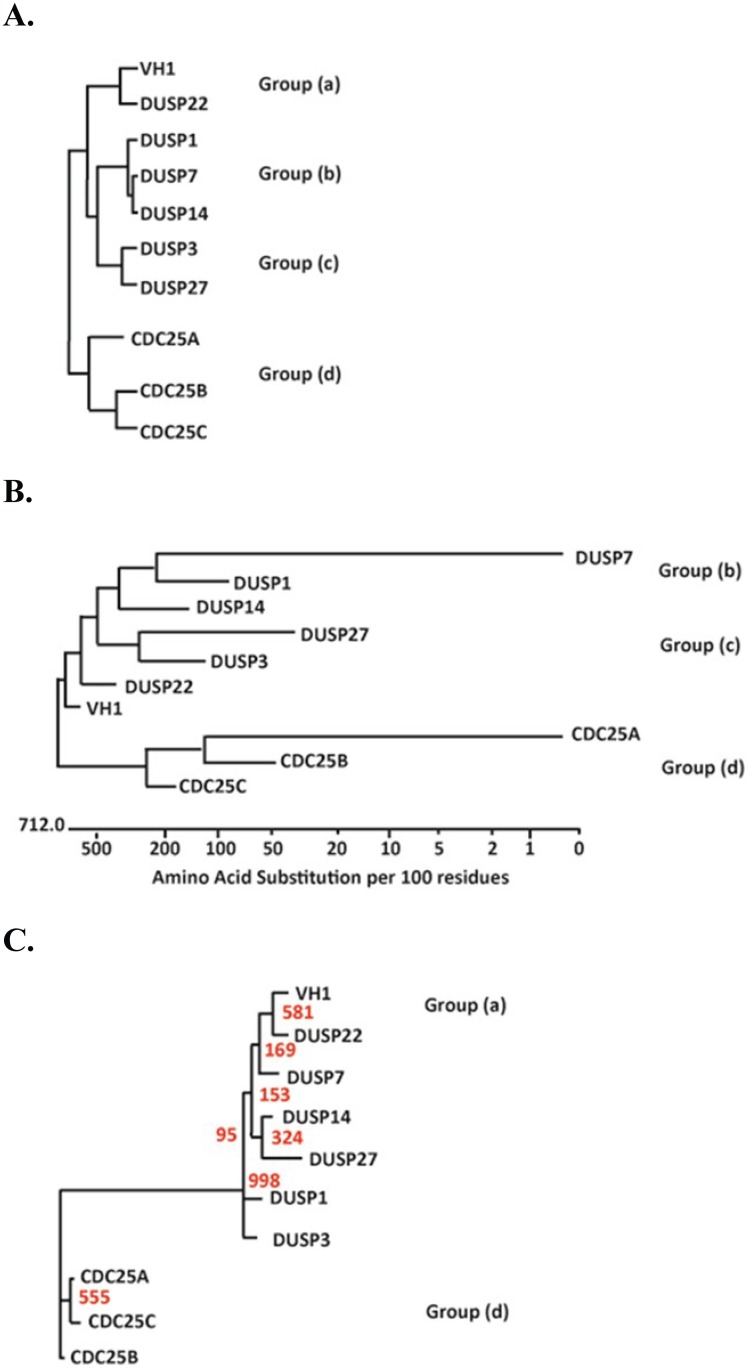
Relationship between catalytic recognition of Tyr(P)- peptides and DUSP phylogeny. (**A**) Hierarchical clustering of DUSPs was performed based on percent dephosphorylation of microarrayed Tyr(P) peptide substrates. (**B**) A cladogram representation of phylogenetic relationship of different DUSPs based on the multiple alignment of the catalytic domain amino acid sequences (Jotun Hein method). (**C**) A conserved region of 15 residues surrounding the active site of each DUSP was used to construct a dendrogram representative of similarity within phosphatase sequences involved in substrate recognition. A maximum likelihood tree is depicted with bootstrap values (out of 1000 replicates) shown in red.

Hierarchical clustering of the Tyr(P) dephosphorylation data (Fig 5A) demonstrated that the DUSPs can be organized into four primary activity clusters based on Tyr(P) peptide substrate specificity: (*a*) VH1 and DUSP22; (*b*) DUSP1, DUSP7, DUSP14, DUSP3 and DUSP27, in which DUSP3 and DUSP27 are closely clustered; (*c*) DUSP1, DUSP7 and DUSP14; (*d*) and a fourth cluster consisting of Cdc25A, Cdc25B and Cdc25C. While each enzyme displayed a unique pattern of substrate specificity, the clustering analysis suggested that DUSPs could be grouped by shared substrates. We first assessed the phylogenetic profiles of 10 DUSPs using the amino acids of the catalytic domain. Phylogenetic trees of the 10 DUSPs were constructed based on multiple sequence alignment methods (Fig 5B). Clusters (b), (c), and (d) in the phylogenetic dendrogram presented in Fig 5 were almost identical to the clusters in the hierarchical clusters (Fig 5A). VH1 and DUSP22 are closely related by phylogeny but did not cluster into the same group (Fig 5B). Assuming that active site residues are the most relevant component of the phosphatase for substrate recognition, we further examined peptide substrates and phosphatase activity by phylogenic relationships. We identified a conserved region within the multiple sequence alignment of DUSPs, consisting of 15 amino acid residues surrounding the catalytic site. The dendrogram constructed from the 15-residue DUSP active site sequence (Fig 5C) exhibited topological features that were very similar to the experimental peptide recognition patterns (Fig 5A). For example, identical peptide substrate and DUSP catalytic site clusters were apparent for Cdc25A-C, as well as VH1 and DUSP22. Collectively, these results suggested a potential relationship between similarities of catalytic sites and substrate recognition motifs.

**Fig 6:**
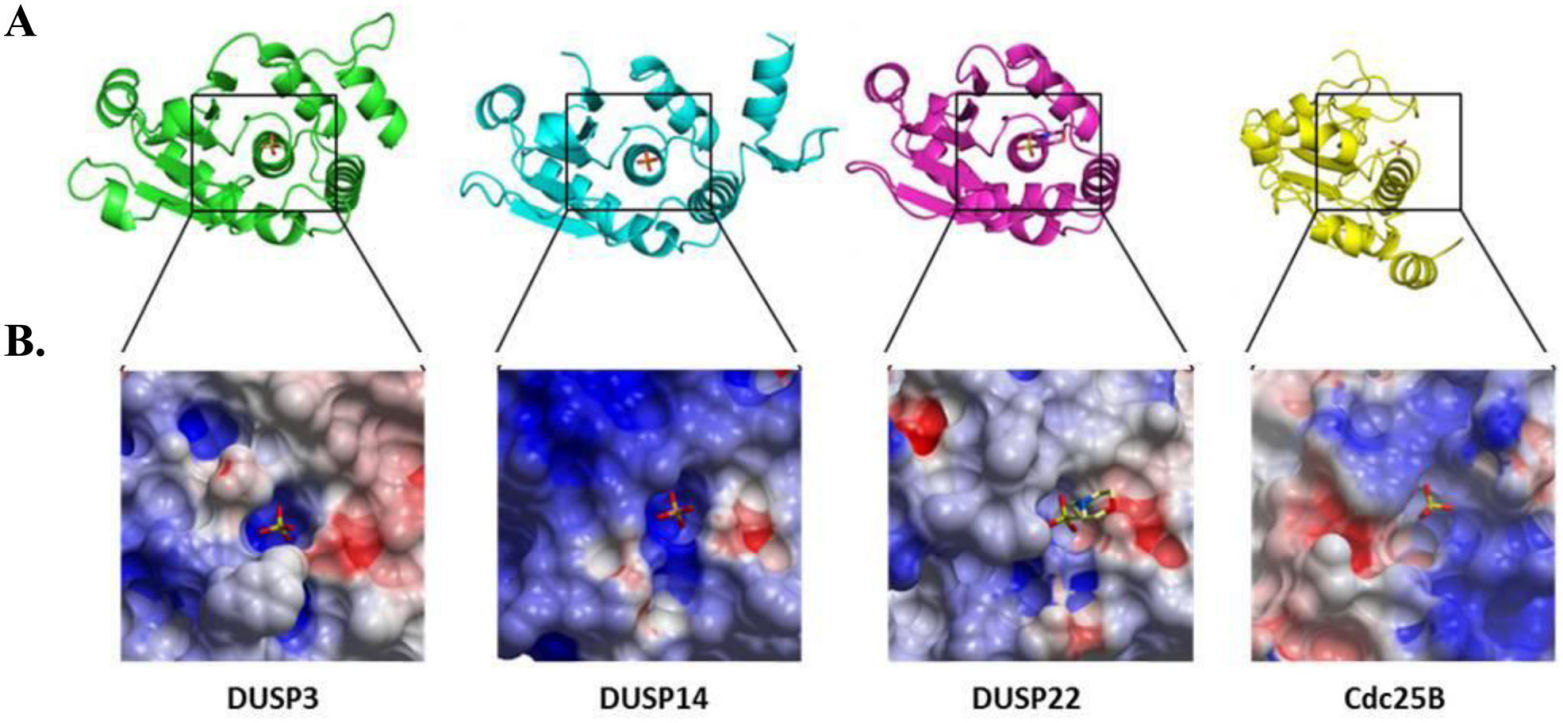
Structural comparison of DUSP3, DUSP14, DUSP22 and Cdc25B catalytic sites. (**A**) Superimposed ribbon representation of structures for DUSP3 (green), DUSP14 (magenta), DUSP22 (cyan) and Cdc25B (yellow). The catalytic sites are centered on the co-crystallized phosphate or 2-(N-morpholino) ethanesulfonic acid (MES) atom. (**B**) Electrostatic potential surface representation of the catalytic site of DUSP3 (PDB: 1VHR), DUSP14 (PDB: 2WGP), DUSP22 (PDB: 1WRM) and Cdc25B (PDB: 1QB0). Red and blue colored regions denote negative and positive charges, respectively.

Further analysis of DUSP surface features suggested possible explanations for the diversity in Tyr(P) peptide recognition. We noted similar peptide substrate motifs for VH1 and the Cdc25s, with a preponderance of acidic residues (Fig 3), suggesting an important role for negative electrostatic potential in substrate docking. Curiously, DUSP14, with the most negatively charged surface surrounding the catalytic site, preferred substrates comprised of neutral or slightly polar residues (Fig 3). The protein structures of the Cdc25A, Cdc25B and Cdc25C catalytic domains are very similar to each other, but most distant from the other DUSPs examined in our study (S2 Table). The molecular structures of DUSPs representative of the four substrate clusters (Fig 5A: DUSP3, DUSP14, DUSP22 and Cdc25B) were further examined for sequence identity, the root-mean-square deviation (RMSD) of atomic position and the Cα-alignment (Q score) (S1 Fig and S2 Table). While the DUSPs we examined have very similar or identical catalytic site sequence motifs (Table 1), the 3-dimensional structures fall into two general folds (Fig 6A). The common alpha helix that is perpendicular to the surface of the catalytic pocket (center of box in Fig 6A) aligned well with the other DUSP structures (DUSP3, DUSP14 and DUSP22). However, to properly align the Cdc25B catalytic site, the orientation of the surface model was slightly shifted in perspective compared to the other structures shown in Fig 6A. In another feature, the electrostatic potential of the surfaces surrounding the catalytic site are distinct for each of the modeled DUSPs (Fig 6B), with several commonalities. All of the DUSP surfaces harbor a positively-charged surface that is near the Tyr(P)-binding pocket. For DUSP3, one surface adjacent to the catalytic site presents a positive electrostatic potential that is flanked on the opposite side of the catalytic site by a large negatively-charged patch. The DUSP14 surface nearest the catalytic site is mostly hydrophobic, while the remaining areas are positively charged. The distribution of surface electrostatic potential for DUSP22 is very similar to DUSP3, with a positively charged region on one side of the catalytic site and a mixed negatively charged or neutral region on the adjacent side. In the case of Cdc25B, a narrow positively-charged area surrounds the Tyr(P) pocket.

## Discussion

We identified optimal substrate sequence motifs for dephosphorylation of Tyr(P) residues by enzymes that are representative of four distinct categories of DUSPs. The substrate motifs identified in our study will be important for examining the structural relationships that drive interactions with cellular targets. In considering the diversity of the most active substrates represented by the Tyr(P) peptide substrates, the enzymatic activities of the DUSPs were directed towards at least 29 signaling pathways, and most significantly for PI3K-Akt, calcium, ErbB, neurotrophin, and chemokine pathways. Although distinct sequence preferences were evident for each DUSP, a high degree of substrate promiscuity was also apparent. It is possible that the DUSPs we examined may interact with multiple substrates during normal or pathological cellular processes, as recently postulated for PTP1B [59]. Substrate-trapping mutants of DUSPs also engage in stable interactions with many native cellular proteins (our unpublished observations). Further, a non-catalytic phosphate-binding pocket that is observed in many PTPs [60] may influence substrate interactions. We note that our results do not directly address catalytic activity directed towards Ser(P) or Thr(P) residues, and that protein-protein interactions occurring outside of the active site also guide the catalytic domain to the correct intracellular substrate.

General conclusions regarding enzyme-ligand interactions are possible based on our results, though further study will be required to confirm that the motifs we identified represent legitimate biological substrates. The most highly dephosphorylated peptides represented proteins from 29 cell-signaling pathways, greatly expanding the list of potential physiological targets. The DUSPs examined in our study fell into four primary activity clusters based on substrate specificity. We examined relationships among DUSPs catalytic activities, catalytic domain sequences and conserved catalytic sites. Based on the relative agreement between the phylogenetic dendrograms, our results suggested a potential relationship between similarities of catalytic sites and substrate recognition motifs.

The DUSPs use a common dephosphorylation mechanism [4] consisting of a thiophosphoryl intermediate that is formed by a thiolate nucleophilic attack of the catalytic site Cys anion directed towards the phosphoryl group of the peptidyl Tyr(P), assisted by an invariant Asp that is located in the P loop in all PTPs except the Cdc25s. Certain features of the peptide motifs we describe and DUSP surfaces reported by others provide clues regarding possible mechanisms for substrate recognition. The predominance of acidic residues flanking the Tyr(P) within the peptide motifs implies that negative surface electrostatic potential is important for substrate docking, while the positive electrostatic surfaces near the DUSP catalytic site may complement the incoming phosphate group. In a similar manner for single-specificity PTPs, negatively-charged residues were favored while positively charged residues were unfavorable for peptide sequence selection [61]. Yet, our results also suggest that DUSPs may be less selective than previously considered. It is possible that the shallow catalytic pockets and relatively flat protein surface features that are characteristic of most DUSPs drives the promiscuous phosphatase activity noted in our study. For example, catalytic domains of Cdc25A-C are extremely shallow and open [36], with no auxiliary loop extending over the active site to facilitate substrate dephosphorylation, and the surface surrounding the catalytic pocket of the poxvirus VH1 is very flat [23].

We considered the potential contribution of ‘dual-specificity’ to our results, as the microarrayed peptide library that we employed contained only Tyr(P) substrates. The Thr(P) binding site was identified in the co-crystal structure of DUSP3 in complex with a biphosphorylated p38 peptide [15], providing direct evidence for dual-specificity substrate docking. The Thr(P) pocket of DUSP3 is partially formed by the positively charged Arg158 residue. The DUSP22 residue Arg122 also forms a positively charged pocket that was postulated to play the same role as Arg158 in DUSP3 [35]. In a previous report, Cdc25s dephosphorylated a Cdk2 peptide containing Thr14(P) and Tyr15(P) residues more efficiently than the same peptide monophosphorylated at either position [62]. The preference for negatively-charged residues at the -1 or +1 position relative to Tyr(P) in the conserved motifs for Cdc25s may mimic the negatively charged Thr14(P) residue of Cdk2 protein. It is possible that the acidic or hydroxyl side chain present in the +2 position relative to Tyr(P) in most but not all of the peptide substrate motifs (Fig 3) substituted for Thr/Ser(P) in substrate recognition by the DUSPs we examined. In addition to the negatively charged amino acid residues, Ser, Thr and Tyr were present in select DUSP peptide substrate motifs. One explanation for this observation is that these residues may bind to the secondary pocket for Thr(P)/Ser(P) hydrolysis, or stabilize the peptide-phosphatase interactions to facilitate dephosphorylation of the Tyr(P) residue, as seen in the Thr(P) reside of p38 peptide binding to the Arg158 pocket on DUSP3 [15]. Combining the newly identified DUSP substrates from our study with optimal Thr(P) or Ser(P) motifs will be important for clarifying these structure-activity relationships and for the design of chemical probes to explore potential biological roles.

## Supporting Information

S1 Fig Multiple amino acid sequence alignment of DUSP catalytic domains. (TIF)

S1 Table Cell signaling pathways associated with DUSP peptide substrates. (DOCX)

S2 Table Sequence identity and 3D structure comparison (RMSD) of DUSP proteins. (DOCX)
