# Supplementary figures and images for: Phosphotyrosine Substrate Sequence Motifs for Dual Specificity Phosphatases
Source: PLoS One. 2015 Aug 24;10(8):e0134984. doi: 10.1371/journal.pone.0134984 (PMC4547750; doi:10.1371/journal.pone.0134984)

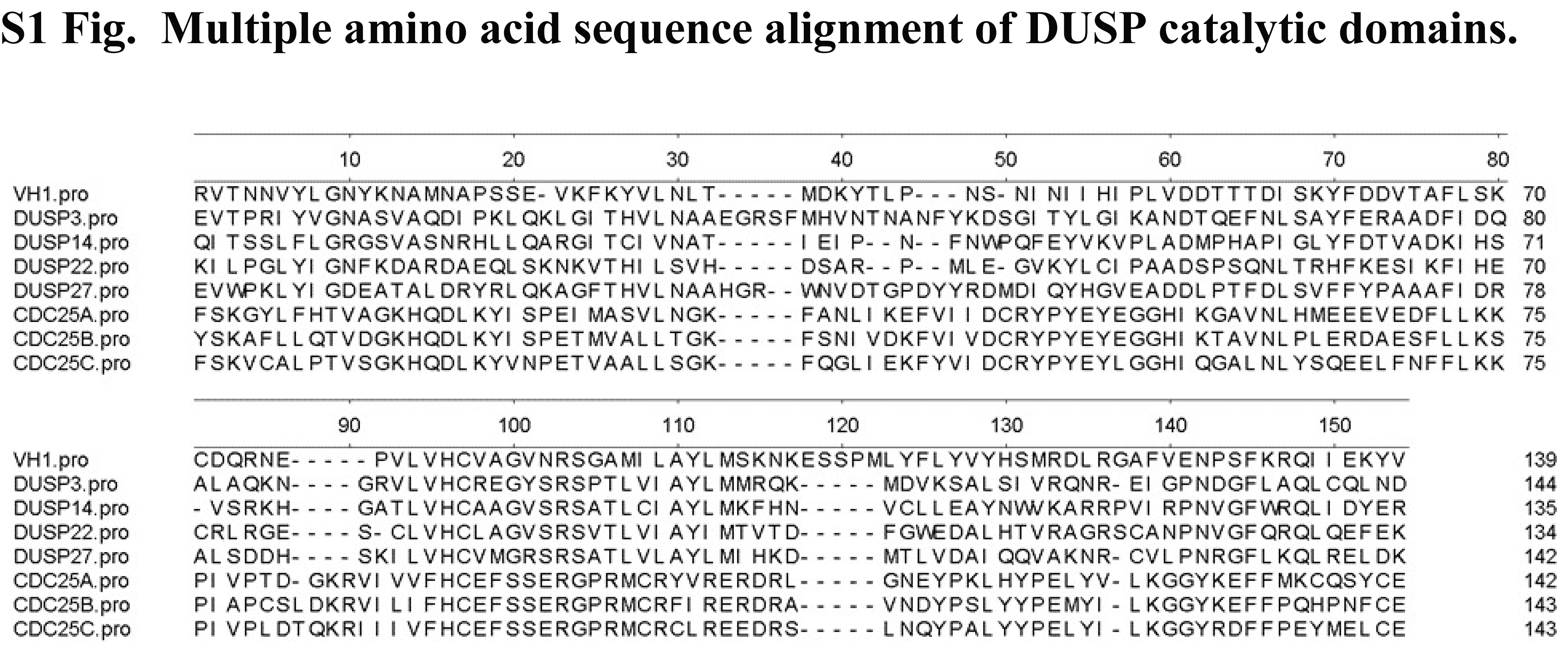

Supplement: S1 Fig — (TIF) [file pone.0134984.s001.tif]
