# Supplementary material for: Phosphotyrosine Substrate Sequence Motifs for Dual Specificity Phosphatases
Source: PLoS One. 2015 Aug 24;10(8):e0134984. doi: 10.1371/journal.pone.0134984 (PMC4547750; doi:10.1371/journal.pone.0134984)
Supplement: S1 Table — (DOCX) [file pone.0134984.s002.docx]

**S1 Table. Cell signaling pathways associated with DUSP peptide substrates.**

| **KEGG Pathway ID** | **Signaling Pathway Description** | **P-value** | **Phosphatase Count** | **Peptide Subset Count** | **Peptide Background Count** |
| --- | --- | --- | --- | --- | --- |
| hsa04151 | PI3K-Akt | *0.00000580* | 10 | 34 | 81 |
| hsa04010 | MAPK | 0.0501 | 10 | 29 | 81 |
| hsa04020 | Calcium | *0.0000100* | 10 | 26 | 39 |
| hsa04722 | Neurotrophin | *0.000129* | 10 | 25 | 42 |
| hsa04062 | Chemokine | *0.0001946* | 10 | 23 | 38 |
| hsa04012 | ErbB | *0.00000100* | 10 | 22 | 25 |
| hsa04066 | HIF-1 | 0.00790 | 10 | 19 | 39 |
| hsa04660 | T cell receptor | 0.281 | 10 | 15 | 46 |
| hsa04630 | Jak-STAT | 0.0323 | 10 | 16 | 36 |
| hsa04910 | Insulin | 0.0157 | 10 | 16 | 33 |
| hsa04064 | NF-κ B | 0.0382 | 10 | 11 | 22 |
| hsa04912 | GnRH | 0.305 | 10 | 12 | 36 |
| hsa04664 | Fc ε RI | 0.309 | 10 | 10 | 29 |
| hsa04620 | Toll-like receptor | 0.377 | 10 | 9 | 27 |
| hsa04115 | p53 | 0.133 | 10 | 8 | 18 |
| hsa04350 | TGF-β | 0.264 | 6 | 7 | 18 |
| hsa04915 | Estrogen | 0.776 | 10 | 8 | 30 |
| hsa04662 | B cell receptor | 0.776 | 10 | 7 | 33 |
| hsa04370 | VEGF | 0.300 | 10 | 8 | 22 |
| hsa04668 | TNF | 0.535 | 10 | 6 | 33 |
| hsa04390 | Hippo | 0.972 | 8 | 6 | 37 |
| hsa04150 | mTOR | 0.171 | 10 | 7 | 16 |
| hsa04917 | Prolactin | 0.810 | 10 | 6 | 28 |
| hsa04310 | Wnt | 0.133 | 10 | 4 | 36 |
| hsa04070 | Phosphatidylinositol | 0.500 | 10 | 5 | 29 |
| hsa04330 | Notch | 0.959 | 5 | 3 | 13 |
| hsa04920 | Adipocytokine | 0.317 | 10 | 7 | 19 |
| hsa04622 | RIG-I-like receptor | 0.291 | 1 | 2 | 18 |
| hsa03320 | PPAR | 0.248 | 8 | 1 | 13 |
