# Supplementary material for: Phosphotyrosine Substrate Sequence Motifs for Dual Specificity Phosphatases
Source: PLoS One. 2015 Aug 24;10(8):e0134984. doi: 10.1371/journal.pone.0134984 (PMC4547750; doi:10.1371/journal.pone.0134984)
Supplement: S2 Table — (DOCX) [file pone.0134984.s003.docx]

**S2 Table. Sequence identity and 3D structure comparison (RMSD) of DUSP proteins.**

| **Sequence Identity** | | | | | | | | |
| --- | --- | --- | --- | --- | --- | --- | --- | --- |
| **Structure (PDB)** | **VH1** | **DUSP3** | **DUSP14** | **DUSP22** | **DUSP27** | **Cdc25A** | **Cdc25B** | **Cdc25C** |
| **VH1** |  | 0.353 | 0.324 | 0.338 | 0.324 | 0.088 | 0.074 | 0.088 |
| **DUSP3** | 0.353 |  | 0.441 | 0.412 | 0.544 | 0.088 | 0.088 | 0.132 |
| **DUSP14** | 0.324 | 0.441 |  | 0.309 | 0.382 | 0.059 | 0.059 | 0.059 |
| **DUSP22** | 0.338 | 0.412 | 0.309 |  | 0.368 | 0.044 | 0.074 | 0.088 |
| **DUSP27** | 0.324 | 0.544 | 0.382 | 0.368 |  | 0.059 | 0.088 | 0.088 |
| **Cdc25A** | 0.088 | 0.088 | 0.059 | 0.044 | 0.059 |  | 0.618 | 0.500 |
| **Cdc25B** | 0.074 | 0.088 | 0.059 | 0.074 | 0.088 | 0.618 |  | 0.485 |
| **Cdc25C** | 0.088 | 0.132 | 0.059 | 0.088 | 0.088 | 0.500 | 0.485 |  |
| **RMSD** | | | | | | | | |
| **Structure (PDB)** | **VH1** | **DUSP3** | **DUSP14** | **DUSP22** | **DUSP27** | **Cdc25A** | **Cdc25B** | **Cdc25C** |
| **VH1** |  | 1.864 | 1.462 | 1.420 | 1.802 | 3.464 | 3.504 | 3.654 |
| **DUSP3** | 1.864 |  | 1.241 | 1.697 | 0.830 | 3.474 | 3.419 | 3.588 |
| **DUSP14** | 1.462 | 1.241 |  | 1.270 | 1.388 | 3.338 | 3.323 | 3.453 |
| **DUSP22** | 1.420 | 1.697 | 1.270 |  | 1.740 | 3.422 | 3.420 | 3.489 |
| **DUSP27** | 1.802 | 0.830 | 1.388 | 1.740 |  | 3.487 | 3.446 | 3.586 |
| **Cdc25A** | 3.464 | 3.474 | 3.338 | 3.422 | 3.384 |  | 1.729 | 2.682 |
| **Cdc25B** | 3.504 | 3.419 | 3.323 | 3.420 | 3.446 | 1.729 |  | 2.364 |
| **Cdc25C** | 3.654 | 3.588 | 3.453 | 3.489 | 3.586 | 2.682 | 2.364 |  |
| **Q-score** | | | | | | | | |
| **Structure (PDB)** | **VH1** | **DUSP3** | **DUSP14** | **DUSP22** | **DUSP27** | **Cdc25A** | **Cdc25B** | **Cdc25C** |
| **VH1** |  | 0.167 | 0.199 | 0.203 | 0.172 | 0.100 | 0.100 | 0.095 |
| **DUSP3** | 0.167 |  | 0.203 | 0.182 | 0.210 | 0.097 | 0.099 | 0.094 |
| **DUSP14** | 0.199 | 0.203 |  | 0.217 | 0.199 | 0.108 | 0.109 | 0.104 |
| **DUSP22** | 0.203 | 0.182 | 0.217 |  | 0.182 | 0.106 | 0.106 | 0.104 |
| **DUSP27** | 0.172 | 0.210 | 0.199 | 0.182 |  | 0.098 | 0.100 | 0.095 |
| **Cdc25A** | 0.100 | 0.097 | 0.108 | 0.106 | 0.098 |  | 0.173 | 0.128 |
| **Cdc25B** | 0.100 | 0.099 | 0.109 | 0.106 | 0.100 | 0.173 |  | 0.143 |
| **Cdc25C** | 0.095 | 0.094 | 0.104 | 0.104 | 0.095 | 0.128 | 0.143 |  |
